# Gum Section

**Published:** 1904-12

**Authors:** O. H. Simpson


Gum Section.—I am ready to admit that gum sections are lacking in some essentials, but between the two evils choose the lesser. The prejudice against them is due largely, I believe, to ignorance of their possibilities. I shall not, in these few lines, attempt to explain, only in a general way, how gum sections may be used instead of pink rubber. For a full denture, gum sections may be used above. All that is lost in occlusion can be overcome by using plain teeth below, but in no instance would I use pink rubber in a lower plate.

It adds nothing to its appearance in the mouth, but detracts from the durability. In the upper plates that are to articulate with the lower natural teeth and where there is no likelihood of the gums being exposed, the molars and sometimes bicuspids can be plain teeth. In such cases as this nothing is lost by using red rubber gums. You thus have a stronger and less clumsy denture. The principal objection to gum sections are the tendency to dark joints, checking, and the accumulation of secretions back of them—all of which can be prevented by proper grinding and waxing.—*Dr. O. H. Simpson, Extract West. Journ.*

